# Feasibility of optimal vertex size and spacing for lattice radiotherapy implementation using helical tomotherapy

**DOI:** 10.3389/fonc.2025.1512064

**Published:** 2025-03-07

**Authors:** Yunji Seol, Young Kyu Lee, Byeong Jin Kim, Kyu Hye Choi, Ji Hyun Hong, Chan-beom Park, Sun Hwa Kim, Hyeong Wook Park, Jung-Il Kim, Wonjoong Cheon, Young-nam Kang, Byung Ock Choi

**Affiliations:** ^1^ Department of Radiation Oncology, Seoul St. Mary’s Hospital, College of Medicine, The Catholic University, Seoul, Republic of Korea; ^2^ Department of Biomedicine & Health Sciences, The Catholic University of Korea, Seoul, Republic of Korea; ^3^ Department of Medical Physics, Kyonggi University, Suwon, Republic of Korea; ^4^ Electro-Medical Device Research Center, Korea Electrotechnology Research Institute, Ansan, Republic of Korea

**Keywords:** lattice radiotherapy, spatially fractionated radiotherapy, helical tomotherapy, vertex, valley-to-peak dose ratio

## Abstract

**Purpose:**

Lattice radiotherapy (LRT), a type of spatially fractionated radiotherapy (SFRT), delivers high dose at specific volumes of lattice structure within the tumor to create a low valley-to-peak dose ratio (VPDR). This study aims to evaluate the feasibility of implementing SFRT using helical tomotherapy and to investigate the effects of vertex size and spacing for attaining the VPDR.

**Methods:**

A three-dimensional lattice structure with 3×3×3 vertices was designed in a cheese phantom. Vertex sizes of 0.5 cm, 1.0 cm, and 2.0 cm were assessed, with spacing from 1.0 cm to 5.0 cm. The prescribed dose was set to 20 Gy to the vertices in a single fraction. VPDR was calculated from dose profiles along lines connecting three vertices in the anterior-posterior (AP), lateral (LAT), and superior-inferior (SI) directions. The minimum, maximum, and mean dose for each vertex, as well as conformity, homogeneity and monitor unit (MU) analysis were also performed.

**Results:**

VPDR decreased significantly with increasing vertex size and spacing. While the AP and LAT directions showed similar VPDR values, the SI direction consistently exhibited lower VPDR values across all configurations. Vertex sizes of 0.5 cm, 1.0 cm, and 2.0 cm required spacing of at least 3.0 cm, 2.0 cm, and 1.0 cm, respectively, to achieve VPDR values below 0.4. The conformity indices ranged from 1.0 to 4.02, and the homogeneity indices ranged from 1.20 to 1.57 across all configurations. Additionally, the MUs increased with both vertex size and spacing.

**Conclusions:**

This study quantitatively analyzed the impact of various vertex sizes and spacings on VPDR in lattice radiotherapy using helical tomotherapy. VPDR decreased with increasing vertex size and spacing, with consistently lower values in the SI direction. These findings provide crucial insights for optimizing LRT plans. The identified relationships between the parameters and VPDR offer a foundation for developing more effective LRT protocols in helical tomotherapy, potentially improving therapeutic outcomes

## 1 Introduction

Spatially fractionated radiotherapy (SFRT) represents a radiation therapy technique that delivers high-dose radiation to generate distinct dose peaks and valleys within a tumor (1). This method effectively targets the gross tumor by creating physical dose contrast between high-dose and low-dose areas, thereby damaging and inhibiting tumor cells (2, 3). Specifically, it could have a therapeutic effect by directly targeting tumor cells in high-dose areas and impacting cells in adjacent low-dose regions.

This technique enables the escalation of radiation doses for large tumors, achieving significant treatment efficacy with minimal toxicity (4). The safety and effectiveness of SFRT have been corroborated by numerous clinical studies and further supported by radiobiological and immunological research findings (5–10). The effectiveness of SFRT is supported by several key radiobiological mechanisms, including the bystander effect (2, 10), which refers to the impact of radiation on non-irradiated cells nearby; vascular injury, which disrupts the tumor’s blood supply; and the stimulation of anti-cancer immune responses (8). These mechanisms collectively enhance the anti-tumor effects of SFRT, making it a potent treatment option for patients with bulky tumors (11, 12).

The initial application of SFRT required the use of custom-made or commercially available GRID blocks attached to linear accelerators. However, the physical GRID blocks introduced challenges in clinical applications: 1) difficulty in accurately calculating and measuring dose distribution, 2) technical complexities and inconvenience, and 3) excessively high dose to the skin and normal tissues traversed by the beam. To address these issues, the concept of lattice therapy (LRT) has emerged, which utilizes multi-leaf collimator (MLC)-based intensity-modulated radiotherapy (IMRT) to achieve 3D spatially fractionated dose distribution without physical GRID blocks (12, 13).

Since the first patient was treated with LRT in 2014, over 150 patients with large tumors have received LRT worldwide (13). A systematic review of LRT was conducted to determine the effectiveness and safety of LRT, leading to the proposal of a set of technical recommendations and guidelines for its clinical implementation (13, 14). Most LRTs have been implemented as volumetric-modulated arc therapy (VMAT) with a medical linear accelerator. Jiang et al. and Brown et al. implemented LRT using CyberKnife (7, 15). F Ertan et al. conducted a dosimetric comparison study of 3D LRT using the fixed cone collimator of the CyberKnife and the MLC of the conventional linear accelerator (16). Several studies have reported that a charged particle such as the proton, or carbon beam with pencil-beam scanning technique provides better dosimetric performance compared to a photon beam (17–19). While charged particle-based LRT faces challenges like high entrance dose and limited beam angles, recent advancements in arc and FLASH techniques show promise for treating bulky tumors, potentially improving dose conformity.

Recent trends in SFRT research aim to further validate clinical efficacy through biological studies and clinical trials (11–14). These studies focus on demonstrating the therapeutic benefits of spatial fractionation in various cancer types, investigating outcomes such as tumor control, survival rates, and quality of life improvements. This research highlights the advantages of SFRT compared to conventional radiotherapy. Researchers are working to optimize treatment planning systems, determining ideal vertex size, spacing, and patient-specific parameters (20, 21). Gaudreault et al. (20) have developed an automated treatment planning approach for LRT, aiming to streamline the process and improve consistency. Zhang et al. (21) proposed a method for lattice position optimization in LATTICE therapy to enhance dose delivery precision. These efforts refine both the clinical evidence base and technical aspects of treatment delivery, seeking to maximize LRT’s therapeutic potential. However, the optimal vertex size and spacing required to achieve the desired VPDR at specific locations have not been clearly established.

Tomotherapy is a representative binary MLC-based IMRT treatment machine widely used in radiation oncology. During treatment, as the gantry rotates 360°, the couch moves inward at a constant speed, resulting in a spiral radiation delivery pattern directly onto the patient. Tomotherapy offers superior conformity, homogeneity, and normal tissue protection compared to conventional IMRT (22). While some institutions have explored GRID therapy using Tomotherapy (23, 24), LRT represents a novel approach not previously investigated. This study is the first to investigate the feasibility of implementing LRT using Tomotherapy. Therefore, this study aims to analyze VPDR based on various vertex sizes and spacings in the context of Tomotherapy-based LRT. By conducting this pioneering research, this study proposes guidelines for selecting suitable vertex size and spacing for clinical application through quantitative analysis.

## 2 Materials and methods

### 2.1 3D lattice design

Computed tomography (CT) images of the cheese phantom were acquired using a SOMATOM go.Open Pro CT scanner (Siemens Healthineers, Germany) with a slice thickness of 1.0 mm. The cheese phantom was selected for its uniform material composition, making it ideal for dose measurement and distribution analysis.

**Figure 1:**
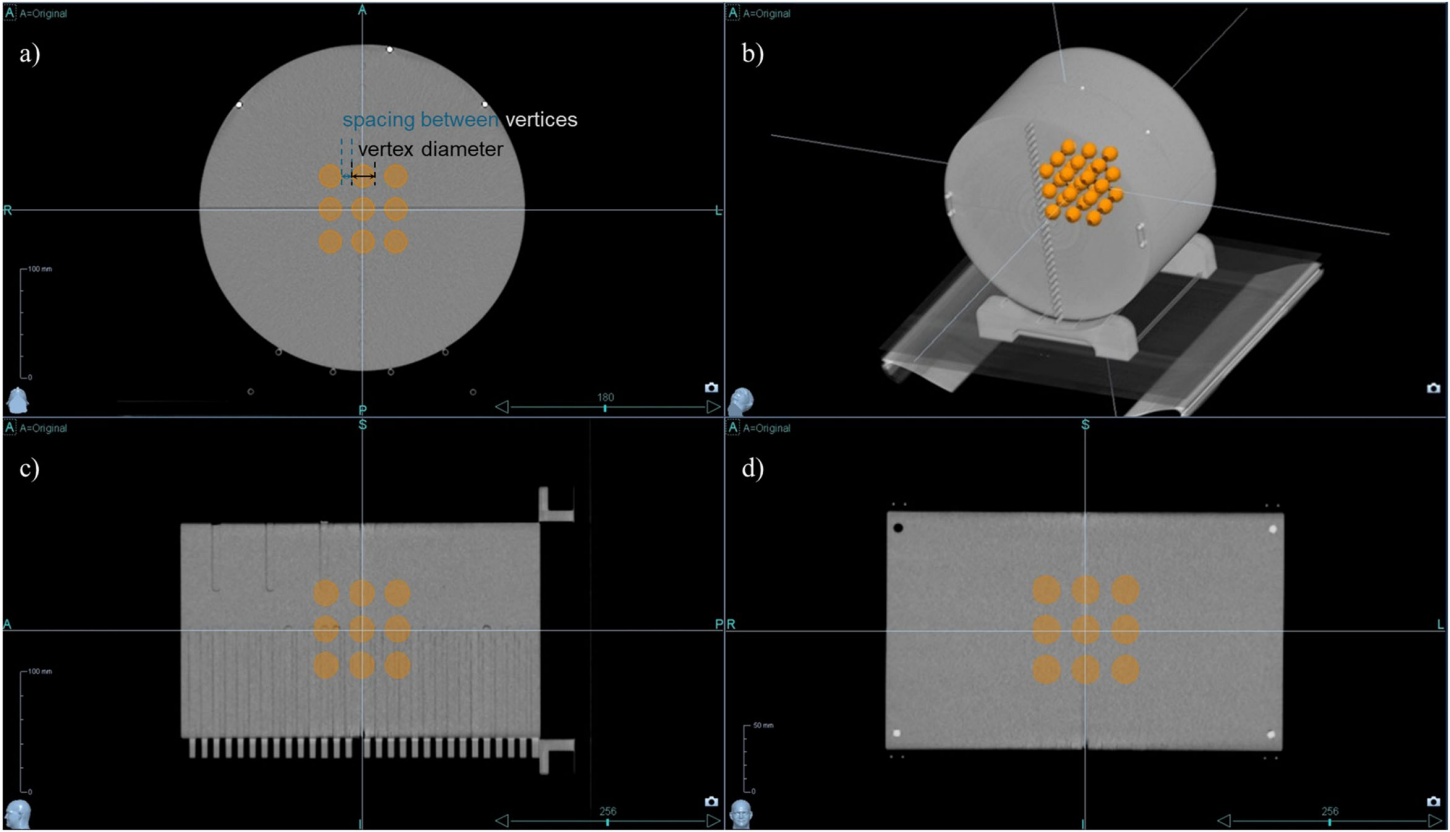
3D lattice structure in cheese phantom CT Images. The designed 3D lattice structure in cheese phantom is displayed in multiple views: **(a)** axial view, **(b)** 3D reconstructed view, **(c)** sagittal view, and **(d)** coronal view. The internal orange spheres represent the vertices of the lattice structure, indicating targeted regions for spatially fractionated dose delivery.

In the central region of the phantom, a 3D lattice structure comprising 3×3×3 vertices was meticulously designed, as shown in **Figure 1**. Each vertex, uniform in size and evenly distributed across the specified area, was integral to our investigation. To determine the optimal dimensions for these vertices, which are crucial for effective treatment planning, we explored a series of configurations. Vertex diameters of 0.5 cm, 1.0 cm, and 2.0 cm were assessed, with the spacing between vertices systematically varied at 1.0 cm, 2.0 cm, 3.0 cm, 4.0 cm, and 5.0 cm. This methodical approach enabled a comprehensive evaluation of the lattice structure’s influence on the spatial dose distribution, laying the groundwork for subsequent analyses aimed at optimizing therapeutic efficacy while minimizing potential adverse effects.

### 2.2 Treatment planning

**Figure 2:**
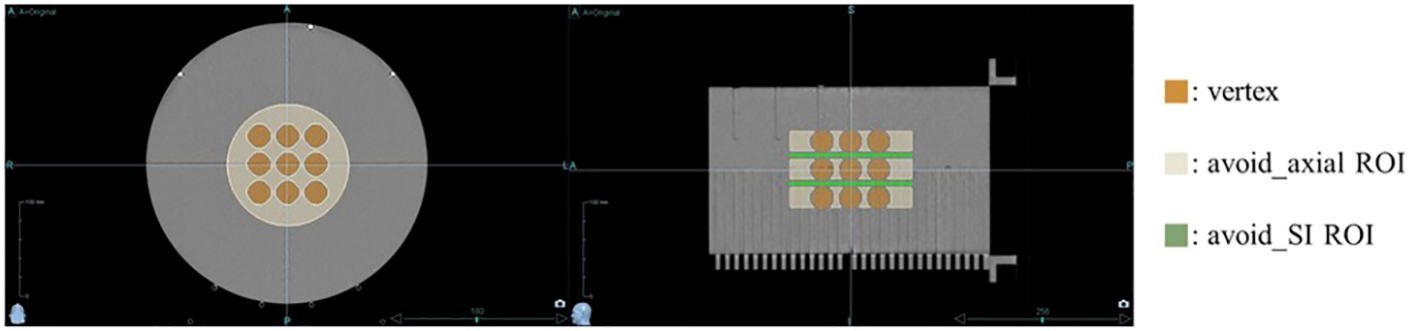
Regions of Interest (ROIs) for optimizing dose distribution. Additional ROIs were specified to optimize dose delivery: the avoid_axial ROI (beige) was designed on the axial slices with a margin, considering the size and spacing of each vertex, while the avoid_SI ROI (green) was set to reduce the dose between vertices along the superior-inferior direction.

Helical tomotherapy plans were executed utilizing the Precision treatment planning system (version 1.1.1.1, Accuray, Sunnyvale, CA, USA). The equipment used for the plan was the Radixact X9 system (Accuray, Sunnyvale, CA, USA) with a dose rate of 1180 MU/min. The prescribed dose was set to 20 Gy in a single fraction, where 50% of the total combined volume of all vertices was intended to receive this dose, with no maximum dose limit imposed within the vertices to enhance the peak dose. Additional planning regions of interest (ROIs) were added to the treatment plan to achieve the desired dose distribution for the lattice structure. As depicted in **Figure 2**, specific ROIs were designated to optimize dose delivery. An avoid_axial ROI was meticulously designed on the axial slice, considering the size and spacing of each vertex, ensuring a customized margin. An avoid_SI ROI was also established to reduce the dose between vertices along the SI direction. Initial settings included a 1.1 cm fixed jaw, a pitch of 0.1, and a modulation factor of 2.0 to achieve a dosimetrically acceptable and clinically deliverable plan. Plan parameters were iteratively adjusted every 50 iterations until the estimated gantry period did not exceed 60.0 s.

### 2.3 Calculate the valley-to-peak dose ratio

**Figure 3:**
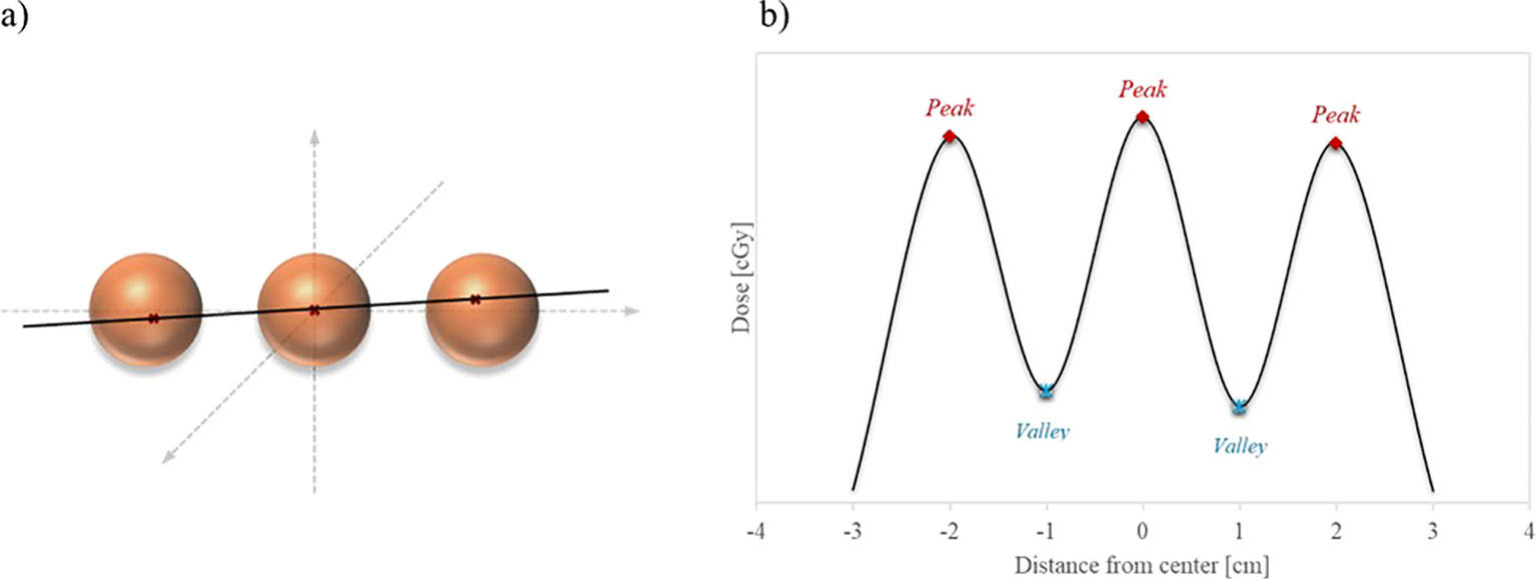
Illustration of dose profile line for VPDR. **(a)** Red crosses within the orange sphere vertices indicate the maximum dose points at each vertex, and the black line represents the closest straight line to these points. **(b)** The extracted dose profile along the black line displays alternating peak doses at the vertices and valley doses between them.


The VPDR value serves as an important indicator for evaluating the dose distribution characteristics of the lattice structure. To calculate the VPDR, the position of the maximum dose point at each vertex was determined. A single line was drawn that most closely approximates and passes near the maximum dose points in three adjacent vertices, and the dose profile along that line was obtained, as illustrated in **Figure 3**. Subsequently, the peak dose ( ${\text{D}}_{\text{peak}}$ ) at the high-dose vertex region and the valley dose ( ${\text{D}}_{\text{valley}}$ ) at the lower-dose region between vertices were identified from the dose profile. The VPDR was then calculated using the following formula:

$$\text{VPDR}=\frac{{\text{D}}_{\text{valley}}}{{\text{D}}_{\text{peak}}}\times 100$$

A total of 27 dose profiles were obtained in the anterior-posterior (AP), lateral (LAT), and superior-inferior (SI) directions, and the average VPDR was calculated for each direction. These VPDR values serve as crucial indicators for evaluating the dose distribution characteristics of the lattice structure and play a pivotal role in guiding efforts for treatment optimization.

### 2.4 Extract plan evaluation metrics

Dosimetric metrics were extracted from the treatment planning system for each configuration to comprehensively evaluate the performance of the treatment plans. Specifically, the minimum, maximum, and mean doses at the 27 vertices, as well as the dose distribution surrounding these vertices, were analyzed. To evaluate the preservation of normal tissue, the ROI excluding the vertices (phantom body – 27 vertices ROI) was derived by subtracting the volume occupied by the vertices from the phantom body contour. The volume percentage of this ROI receiving at least 50% and 30% of the prescribed dose (V50% and V30%, respectively) was extracted and compared across different vertex configurations. Additionally, the conformity index (CI) and homogeneity index (HI) were calculated using dose-volume histogram (DVH) data to assess the precision and uniformity of the dose distribution.

To further evaluate the efficiency and feasibility of LRT using tomotherapy, we analyzed the planned MUs and gantry period time. These parameters were used to assess the overall treatment delivery time and the operational efficiency of the plan.

## 3 Results

### 3.1 Valley-to-peak dose ratio

**Figure 4:**
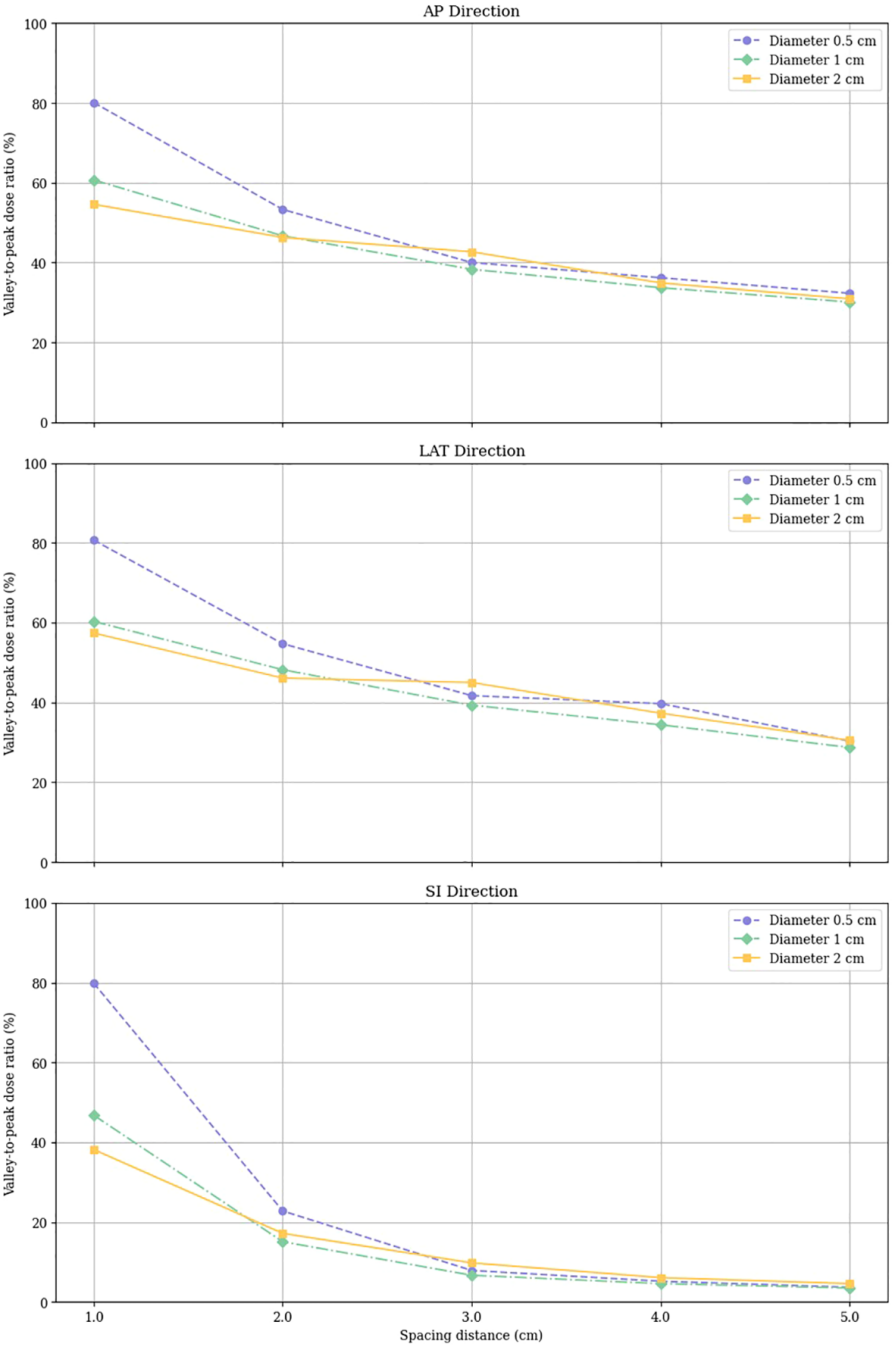
Valley-to-Peak Dose Ratios (VPDR) for various vertex sizes and spacing distances in three directions (AP, Anterior-Posterior; LAT, Lateral; SI, Superior-Inferior).

The VPDR for each configuration of vertex size and spacing was measured along the AP, LAT, and SI directions, and the results are depicted in **Figure 4**. These results indicate that larger peak sizes and increased spacing generally result in higher VPDR values, suggesting a more pronounced dose contrast between peaks and valleys.

The data points representing VPDR corresponding to the spacing between vertices are connected by lines: purple circles for 0.5 cm diameter vertices, green diamonds for 1.0 cm diameter vertices, and yellow squares for 2.0 cm diameter vertices.

VPDR values in the AP and LAT directions showed similar trends and magnitudes. For the 0.5 cm diameter vertices, VPDR ranged from 80.1% to 32.3% in the AP direction and from 80.7% to 30.3% in the LAT direction, with VPDR decreasing as the distance increased from 1.0 cm to 5.0 cm. For the 2.0 cm diameter vertices, VPDR ranged from 54.6% to 30.9% in the AP direction and from 57.4% to 30.5% in the LAT direction over the same distance range. VPDR values in the SI direction were consistently lower compared to those in the AP and LAT directions. For 0.5 cm diameter vertices, VPDR in the SI direction ranged from 79.9% at 1.0 cm to 3.7% at 5.0 cm. For the 2.0 cm diameter vertices, the range was from 38.2% to 4.6%.

### 3.2 Dose distribution analysis

Analysis of lattice radiotherapy (LRT) configurations using helical tomotherapy revealed significant impacts of vertex size and spacing on dose distribution, conformity, and homogeneity.

**Figure 5:**
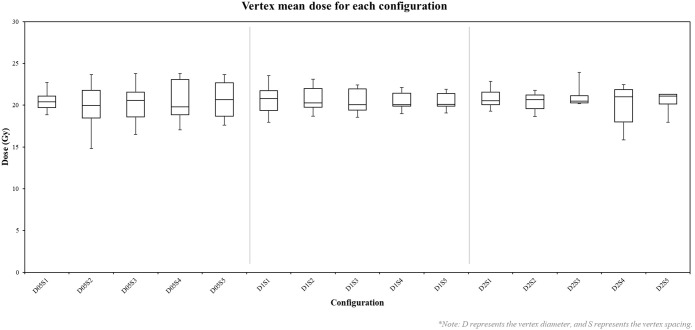
Box plot of vertex mean doses in each configuration. D represents the vertex diameter, and S represents the vertex spacing. Each box represents the distribution of mean doses across 27 vertices for a specific configuration.

Mean doses delivered to vertices ranged from 14.81 Gy to 23.92 Gy, with most vertices receiving doses within ± 2 Gy of the prescribed 20 Gy. As shown in **Figure 5**, configurations with smaller vertex diameters (0.5 cm) exhibited greater variability in mean doses compared to larger diameters. Increasing the spacing between vertices generally resulted in a slight reduction in dose variability within each diameter group. The minimum doses across all configurations ranged from 13.65 Gy to 22.31 Gy, while maximum doses spanned from 15.41 Gy to 31.39 Gy. Notably, configurations with smaller vertex diameters tended to produce higher minimum doses, whereas those with larger diameters exhibited higher maximum doses.

**Figure 6:**
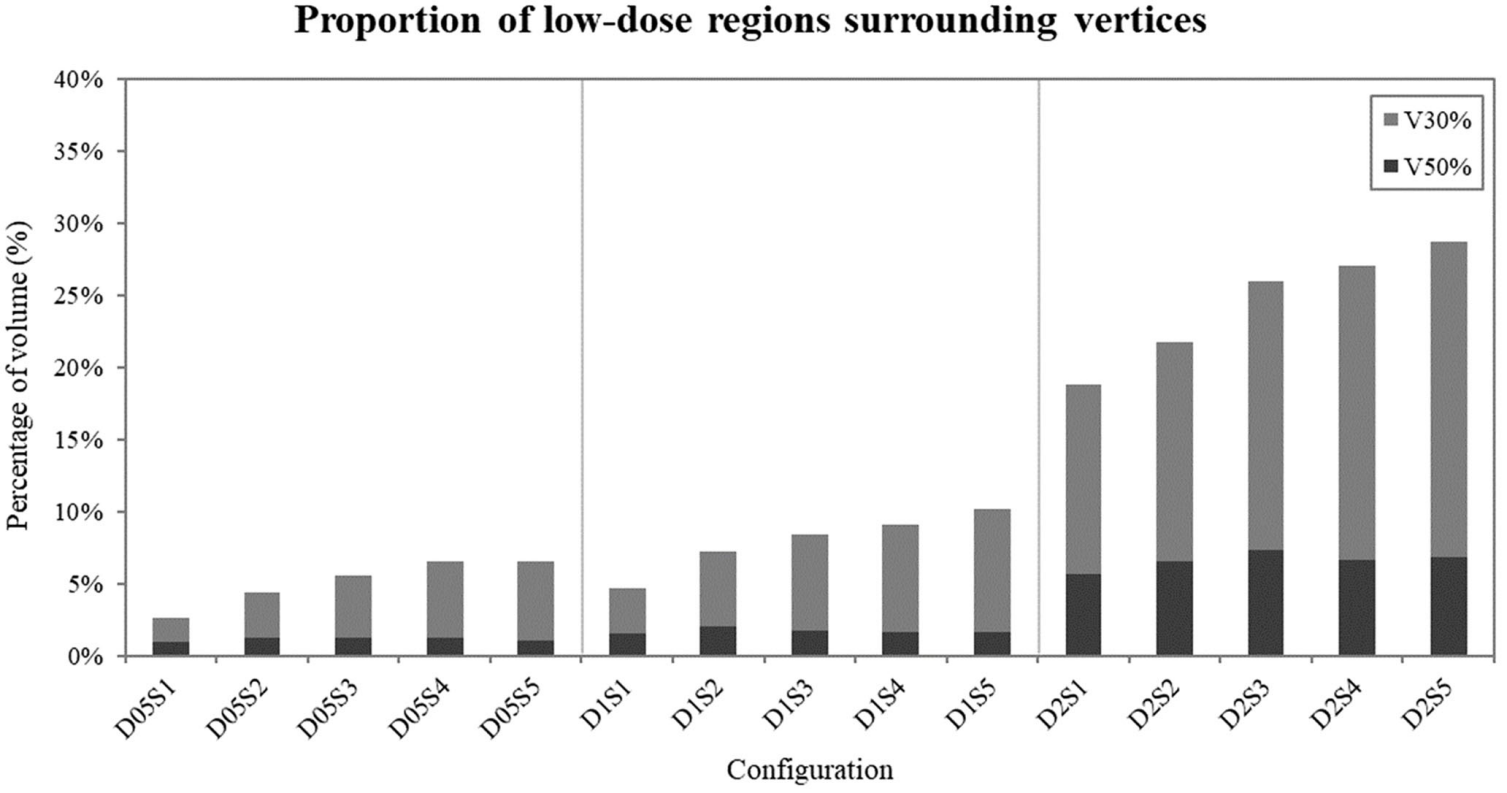
Proportion of low-dose areas (30% and 50% of the prescribed 20 Gy dose) surrounding the vertices in each configuration. Dark bars represent V50% values, while light bars represent V30% values.

Low dose analysis in regions excluding the vertices, illustrated in **Figure 6**, revealed that narrower spacing resulted in lower V30% and V50% values, particularly for configurations with small vertex diameters. Conversely, wider spacing led to higher percentages of V30% and V50% across all vertex diameters, indicating that increasing spacing facilitates achieving the desired VPDR.

The CI, measuring how well the prescribed isodose conforms to the target volume, ranged from 1.00 to 4.02. Configurations with 2.0 cm diameter vertices generally demonstrated better conformity (CI range: 1.00-1.09) compared to smaller diameters. The Homogeneity Index (HI), assessing dose uniformity within the target volume, ranged from 1.20 to 1.57. Configurations with smaller vertex diameters (0.5 cm) generally resulted in better homogeneity (HI range: 1.20-1.26), while larger vertex diameters (2.0 cm) showed slightly poorer homogeneity (HI range: 1.39-1.57).

These findings suggest a trade-off between dose homogeneity and conformity in LRT configurations. While smaller vertex diameters offer better dose homogeneity, larger diameters provide better dose conformity.

### 3.3 Treatment plan delivery metrics

**Table 1** presents the treatment plan delivery metrics for various vertex diameter and spacing configurations. The results demonstrate a clear trend: as vertex diameters and spacing distances increase, treatment delivery parameters become more complex and extended. Planned MU showed a significant increase from 36283.1 for the smallest configuration (0.5 cm diameter, 1.0 cm spacing) to 159448.7 for the largest configuration (2.0 cm diameter, 5.0 cm spacing).

**Table 1:** Treatment plan delivery metrics for different vertex configurations.

Diameter (cm)	Spacing (cm)	Planned MU	Sinogram Segments	Gantry Period (s)	Gantry Rotations	Beam on Time (s)	Couch Travel (mm)
0.5	1	36283.1	14.2	48.8	42.6	2079.3	44.7
	2	54894.6	20.6	50.9	61.7	3145.8	64.8
	3	76467.6	27	54.1	80.9	4382.1	84.9
	4	96843.6	33.4	55.4	100.1	5549.8	105
	5	115211.6	39.8	55.3	119.3	6602.4	125.1
1	1	59061.9	19	59.4	57	3384.6	59.7
	2	78008.6	25.4	58.7	76.1	4470.4	79.8
	3	98170.6	31.7	59	95.2	5625.8	99.9
	4	118194.5	38.2	59.1	114.5	6773.3	120.1
	5	137423.6	44.5	58.9	133.6	7875.3	140.1
2	1	85975.5	28.6	57.5	85.6	4927	89.8
	2	103319.6	35	56.4	105	5920.9	110
	3	129301.4	41.4	59.7	124	7409.8	130.1
	4	147318.7	47.8	58.9	143.2	8442.3	150.2
	5	159448.7	54.2	56.2	162.6	9137.5	170.4

Beam-on time, a factor for patient comfort and treatment efficiency, exhibited a direct correlation with both vertex diameter and spacing, ranging from 2079.3 s to 9137.5 s for the smallest and largest configurations, respectively. The number of gantry rotations increased substantially from 42.6 to 162.6 as vertex size and spacing increased, while gantry period remained relatively stable, ranging from 48.8 to 59.7 seconds across all configurations.

Couch travel distance also increased from 44.7 mm to 170.4 mm, reflecting the larger treatment volumes required for more spread-out configurations. These findings suggest that while larger vertex sizes and spacings may offer dosimetric advantages, they result in increased treatment complexity and delivery time.

## 4 Discussion

This study aimed to demonstrate the feasibility of implementing Lattice Radiotherapy (LRT) using helical tomotherapy and to investigate the impact of vertex size and spacing on treatment planning effectiveness. The evaluation of LRT was based on dosimetric parameters, dose distribution characteristics, and operational metrics that align with the study’s objectives. Specifically, we analyzed the minimum, maximum, and mean doses at the vertices to assess dose uniformity and modulation. Normal tissue sparing was evaluated through V30% and V50%, which represent the percentage of the phantom body (excluding the vertices) receiving at least 30% and 50% of the prescribed dose, respectively. The Conformity Index (CI) and Homogeneity Index (HI) were calculated from dose-volume histograms to measure the precision and consistency of dose delivery. Additionally, operational efficiency was assessed using planned monitor units (MUs) and gantry period time to evaluate the practicality of treatment delivery. These metrics collectively provide a comprehensive framework for assessing the feasibility and effectiveness of LRT in clinical applications, providing crucial insights for balancing the competing objectives of target coverage, dose uniformity, and sparing of surrounding tissues in LRT treatment planning.

Our results indicate that the VPDR, a critical parameter in LRT, decreases exponentially with increasing vertex size and spacing. Quantitatively, we observed that VPDR values ranged from 2.87% to 80.7% across different configurations used in this study, with the highest values achieved with smaller vertex diameters (0.5 cm) and larger spacings (5 cm). Interestingly, we found a consistent anisotropy in dose distribution, with VPDR values in the SI direction approximately 15-20% lower than those in the AP and LAT directions. This directional variation, attributed to the helical delivery pattern of tomotherapy, aligns with findings from previous studies on helical delivery techniques but presents unique considerations for LRT implementation.

The analysis of dose distribution revealed important trade-offs in LRT planning. Smaller vertex diameters (0.5 cm) resulted in better dose homogeneity within the vertices (Homogeneity Index range: 1.20-1.26) but showed greater variability in mean doses (± 2 Gy from prescribed 20 Gy). Conversely, larger vertex diameters (2.0 cm) demonstrated better dose conformity (Conformity Index range: 1.00-1.09) but slightly poorer homogeneity (HI range: 1.39-1.57). These findings are consistent with previous LRT studies using other delivery techniques, such as those reported by Gaudreault et al. (20), but our study uniquely quantifies these trade-offs in the context of helical tomotherapy.

The clinical significance of VPDR has been extensively studied in recent years, with mounting evidence supporting its crucial role in treatment outcomes. VPDR serves as a critical metric in LRT that directly influences the balance between tumor control and normal organ sparing. Studies by Wu et al. (13) demonstrated that lower VPDR values (<0.3) correlate with improved local tumor control rates, particularly in bulky tumors exceeding 5cm in diameter. Jiang et al. (7) reported significant tumor response rates (>60%) in advanced cases when achieving optimal VPDR values while maintaining acceptable toxicity profiles. Furthermore, retrospective analyses by Iori et al. (14) suggest that carefully modulated VPDR can reduce radiation-induced complications in cases where tumors are adjacent to critical organs, with complication rates decreasing by up to 30% compared to conventional approaches. Recent immunological studies by Kanagavelu et al. (8) have revealed that optimal spatial dose variation can enhance anti-tumor immune responses, with evidence of increased tumor-infiltrating lymphocytes in regions receiving peak doses.

Evaluation of low-dose exposure in regions excluding the vertices provided valuable insights into normal tissue sparing. We observed that V30% and V50% values increased by approximately 5-10% for every 1 cm increase in spacing, indicating that while larger spacing may improve VPDR, it also results in greater dose distribution to surrounding tissues. This observation extends the findings of previous studies on normal tissue sparing in LRT and highlights the importance of careful spacing selection in treatment planning.

The lowest maximum dose of 15.41 Gy observed in the D0.5S2 configuration reflects challenges associated with edge vertices. Edge vertices are more prone to underdosing due to reduced beam overlap and limited modulation flexibility at the periphery of the lattice structure, especially in configurations with smaller vertex sizes and narrower spacing. These results highlight the complex interplay between vertex size, spacing, and helical tomotherapy delivery characteristics, which should be carefully considered in future optimization strategies.

The trade-off between potential dosimetric benefits and treatment efficiency is evident in our treatment delivery metrics, where beam-on time increased from 2079.3 s to 9137.5 s as we moved from the smallest to the largest configuration. This significant increase in treatment time should be carefully considered in the planning process to balance therapeutic efficacy with patient comfort and clinical practicality, a consideration that has been less emphasized in previous LRT studies.

Based on our experimental results and clinical considerations, we propose specific guidelines for clinical implementation. Our study indicates that vertex size and spacing selections should be tailored to specific clinical scenarios, with clear correlations between these parameters and treatment outcomes. For most clinical applications, our data suggest that a 1 cm vertex diameter with 3-4 cm spacing offers an optimal balance, resulting in VPDR values below 0.4 while maintaining practical treatment delivery times and acceptable low-dose spread. However, this baseline configuration should be adapted according to tumor characteristics and anatomical considerations.

Specifically, for tumors in proximity to critical structures within 2 cm, increasing vertex spacing to 4-5 cm is recommended to reduce low-dose exposure to surrounding tissues. This configuration maintained therapeutic peak doses while reducing the cumulative dose to adjacent normal tissues by approximately 25%. Conversely, for large (>8 cm), radioresistant tumors, our data support using 2 cm vertex diameter with 1-2 cm spacing to enhance tumor control, though practitioners should be mindful of the associated increase in treatment time, which is approximately 2.5 times longer. Tumor morphology should also guide vertex distribution. For irregularly shaped tumors, our conformity index data suggest that adapting vertex placement to follow tumor contours improves dose conformity by up to 15%. The observed anisotropic VPDR distribution in tomotherapy requires particular attention for tumors extending along the superior-inferior axis, as our results show a consistently lower VPDR in this direction, with a reduction of 15-20% compared to other orientations. This characteristic should be carefully considered in treatment planning, as optimizing delivery in this direction could enhance therapeutic outcomes.

Looking toward future developments, our findings suggest several promising directions for advancing LRT implementation. Integration of automated planning algorithms could optimize vertex placement based on our established VPDR-outcome correlations, while functional imaging-based vertex selection could further personalize treatment by targeting radioresistant regions identified through biological imaging. These approaches, combined with our quantitative understanding of vertex parameter relationships, will be essential in achieving an optimal balance between tumor control and normal tissue sparing, particularly in challenging clinical scenarios.

It is also worth noting that our study used a simplified 3×3×3 lattice structure in the center of a phantom, which may limit its direct clinical applicability. In real clinical scenarios, tumors are often irregular in shape and size, constraining the number and placement of vertices. Additionally, lattice vertices are often offset in adjacent CT slices to minimize overlap of high-dose regions and achieve a more uniform dose distribution across the gross tumor volume.

While our study establishes the technical feasibility and dosimetric characteristics of tomotherapy-based LRT using systematic vertex arrangements, clinical implementation should consider tumor biological factors. Recent studies have demonstrated significant advances in biology-guided vertex placement strategies, including the use of functional imaging (25) and metabolic mapping (26) to identify optimal vertex locations. Areas of high metabolic activity or specific radioresistant regions might benefit more from high-dose vertices compared to necrotic areas. Furthermore, emerging evidence suggests that random vertex distribution patterns may be as effective as systematic arrangements, challenging traditional assumptions about optimal lattice geometry (27).

These findings highlight that future implementations of tomotherapy-based LRT should consider integrating both technical delivery capabilities and biological tumor characteristics. Development of treatment planning approaches that combine our dosimetric findings with functional imaging could lead to more personalized and effective treatment strategies. Future studies should explore automated planning approaches that incorporate offsetting and consider more complex, patient-specific lattice designs to implement LRT using tomotherapy in clinical cases. This approach aligns with recent developments in the field, such as the automated planning method introduced by Gaudreault et al. (20), which could potentially address the complexities of individualized treatment planning in LRT.

In conclusion, while our study demonstrates the feasibility of LRT using helical tomotherapy and provides valuable insights into the effects of vertex size and spacing on treatment parameters, further research is needed to translate these findings into clinical practice. This study builds upon and extends previous work in the field of LRT, offering unique insights into its implementation using helical tomotherapy. Evaluating the efficacy and toxicity of more sophisticated, patient-specific designs in a clinical setting will be crucial for determining the true potential of LRT in diverse patient populations and for advancing the field of spatially fractionated radiotherapy.

## 5 Conclusion

This study demonstrates the feasibility of implementing LRT using helical tomotherapy and provides guidelines for optimizing vertex size and spacing for effective treatment planning. We identified the complex interactions between various dose parameters and treatment delivery considerations, offering a foundational framework for further optimization studies aimed at clinical application. To bridge the gap between experimental results and clinical practice, future research should focus on integrating patient-specific parameters, such as GTV and surrounding critical organs, into lattice placement planning. Additionally, evaluating the clinical outcomes of tumor control and normal tissue toxicity will be crucial for translating these findings into practical use. These efforts could improve LRT treatment techniques and potentially enhance outcomes for patients with large tumors.

## Data Availability

The original contributions presented in the study are included in the article/supplementary material. Further inquiries can be directed to the corresponding author/s.
